# Intraoperative Decision-Making and Technical Aspects of Parathyroidectomy in Young Patients With MEN1 Related Hyperparathyroidism

**DOI:** 10.3389/fendo.2018.00618

**Published:** 2018-10-16

**Authors:** Priscilla F. Nobecourt, Jonathan Zagzag, Elliot A. Asare, Nancy D. Perrier

**Affiliations:** ^1^Department of Surgery, University of Texas Medical Branch, Galveston, TX, United States; ^2^Department of Surgical Oncology, University of Texas MD Anderson Cancer Center, Houston, TX, United States

**Keywords:** hyperparathyroidism, MEN1, subtotal parathyroidectomy, technique, hypercalcemia, decision making, parathyroidectomy, recurrence

## Abstract

One in 5,000 to 1 in 50,000 births have multiple endocrine neoplasia type 1 (MEN1). MEN1 is a hereditary syndrome clinically defined by the presence of two of the following endocrine tumors in the same patient: parathyroid adenomas, entero-pancreatic endocrine tumors and pituitary tumors. Most commonly, patients with MEN1 manifest primarily with signs and symptoms linked to primary hyperparathyroidism. By age 50, it is estimated that 100% of patients with MEN1 will have been diagnosed with primary hyperparathyroidism. These patients will need to undergo resection of their hyperfunctioning glands, however there is no clear consensus on which procedure to perform and when to perform it in these patients. In this original study we describe and explain the rational of our peri-operative approach and management at MD Anderson Cancer Center of MEN1 patients with hyperparathyroidism. This protocol includes preoperative evaluation, intraoperative decision-making and detailed surgical technique adopted for these patients' care. Additionally we review follow-up and disease management in instances of recurrent primary hyperparathyroidism in patients with MEN1 syndrome.

## Introduction

### Definitions

Multiple endocrine neoplasia type 1 (MEN1) is a hereditary syndrome with an autosomal dominant pattern of inheritance. It is characterized by a mutation in the *MEN1* gene on chromosome 11q13 (1). The *MEN1* gene encodes the *menin* protein which plays a crucial role in transcriptional regulation as a co-repressor or co-activator, cell cycle regulation, DNA repair, cell signaling, cytoskeletal structure, cell adhesion and cell motility.

MEN1 syndrome can be difficult to define as it is responsible for combinations of presentation of over 20 different endocrine and non-endocrine tumors. According to the most recent MEN1 Clinical Practice Guidelines (2) there are three ways to diagnose MEN1. The clinical diagnosis of this syndrome is done by diagnosing 2 of the 3 major MEN1 related endocrine tumors in the patient, which are parathyroid adenomas, entero-pancreatic endocrine tumors, and pituitary tumors. It is considered a familial MEN1 if beyond the index patient with a clinical diagnosis of MEN1, a first-degree relative is diagnosed with at least one of the main MEN1 associated tumors. The genetic diagnosis of MEN1 is made in a patient who has a germline mutation in the *MEN1* gene but may be asymptomatic and has not manifested evidence of tumor presence (2).

### Epidemiology

The incidence of MEN1 syndrome is estimated at 1 in 30,000 births (3, 4). Primary hyperparathyroidism (PHPT) is the most common endocrinopathy in patients with MEN1 syndrome and nearly 100% of patients present with PHPT by age 50 (2). The female to male ratio of PHPT among patients with MEN1 syndrome is 1:1 and only 2–4% of patients with PHPT have MEN1 syndrome.

### Clinical presentation and diagnosis

Symptoms associated with PHPT tend to be the first clinical manifestation of MEN1 syndrome and usually present at age 20–25 (5, 6). Hypercalcemia associated symptoms, such as weakness, fatigue, constipation, bone pain, difficulty with concentration, depression, sleep disorders or decreased social interaction are some of the clinical manifestations (7). In many instances, the first sign of PHPT in a patient with MEN1 syndrome is hypercalcemia on routine laboratory work with concomitant non-suppressed (i.e., inappropriately normal) or elevated intact PTH levels in a patient <30 years old.

MEN1 patients with PHPT tend to have multigland disease at presentation (8). There is an asymmetric and asynchronous multigland growth in these patients and the pathology of the parathyroid glands frequently reveals hypercellular parathyroid gland (9).

The diagnosis of PHPT is made biochemically through high normal or elevated serum calcium levels and concomitant inappropriate elevation of intact serum PTH levels. A high index of suspicion for MEN1 syndrome should arise when there is young age at presentation of PHPT, multigland disease, family history or presence of another endocrine tumor. All patients with suspected MEN1 syndrome should undergo genetic testing. Additionally, according to the last MEN1 Guidelines (2), genetic testing should be offered to every patient diagnosed with MEN1 and their first-degree relatives whether they are symptomatic or asymptomatic. Germline mutation testing should always be preempted by genetic counseling and it should be offered to relatives prior to undergoing any other screening (2) if a germline *MEN1* mutation has been identified. Genetic testing may help identify at risk family members so that they may receive early treatment.

Imaging is not necessary for the diagnosis of PHPT. During surgery, as discussed later on, all four parathyroid glands will be explored limiting the role of preoperative imaging as well (5).

## Indications for treatment

The treatment of choice for MEN1 associated PHPT is parathyroidectomy. Due to the presence of multigland disease in this patient cohort, there is a high risk of persistent or recurrent hyperparathyroidism (4). The inability to recognize multigland pathology in these patients leads to performing an incomplete resection of all affected parathyroid glands and is associated with a risk of post-operative persistent or recurrent PHPT (6, 10).

There is a fine line between the complexity of crafting the residual tissue to avoid rapid recurrence which would make for multiple reoperations during the individual patient's lifetime vs. overtreatment which would render the patient aparathyroid commanding lifelong calcium replacement therapy. Weighing the risk-benefit ratio of recurrence, the timing of surgery as well as measurement of outcomes and type of procedure is key in the management of patients with MEN1 syndrome.

Whether patients are symptomatic or asymptomatic, parathyroidectomy is indicated if their (i) calcium level is 1 mg/dL above upper normal range and (ii) they have simultaneous non-suppressed PTH levels (11). In patients that do not meet these criteria but have been diagnosed with PHPT and present with signs and symptoms of hypercalcemia, such as loss of bone mineral density, nephrolithiasis, worsening kidney function, pathological fractures and neurocognitive symptoms have strong indications for parathyroidectomy (11). When a patient with a known *MEN1* mutation presents with no symptoms of hypercalcemia and does not meet the biochemical criteria for parathyroidectomy as mentioned above, there is no level of evidence to support the timing of surgical intervention. Some experts recommend waiting for symptomatic hypercalcemia to manifest itself while monitoring the patient closely (2). This is based on the long-term management strategy of avoiding multiple cervical explorations which pose a higher risk of nerve injury and hypoparathyroidism earlier in life. Additionally, some suggest that waiting for the disease to progress leads to more enlargement of the hypercellular glands allowing them to be more easily identifiable intraoperatively (5, 12). Nonetheless it has been found that decreased bone mineral density manifests itself early on in the disease process and does not correlate with serum calcium levels. It has also been shown that even after parathyroidectomy, restoration of age appropriate bone mass is unobtainable if osteopenia is advanced, hence the recommendation of performing early parathyroidectomy in MEN1 patients (13). In a comparative review at MD Anderson Cancer Center (MDACC) between patients with sporadic and MEN1 associated hyperparathyroidism, Silva et al. observed early bone loss in the fourth decade of life in MEN1 patients with hyperparathyroidism as well as increased bone loss at the lumbar spine, femoral neck and total hip compared to the other group (14). This suggests that the deleterious effects of hyperparathyroidism occur well before other clinical manifestations appear.

At MDACC, we favor early parathyroidectomy in patients with known MEN1 syndrome with the goal to limit fewer downstream disabilities in our patients. It has been shown that bone mineral density loss and damage to the kidneys are processes that start early on in the disease progression even though patients may be asymptomatic (15, 16). Additionally many patients considered asymptomatic manifest with neurocognitive symptoms and parathyroidectomy improves their quality of life (11). Nonetheless, the timing of surgery for each patient is individualized, taking into account other pathologies they may present with and the overall wishes of the patient and his/her family. For those wishing to postpone parathyroidectomy, the ability to accept monitoring in the short and long term must also be taken into account. These patients will require biannual measurement of their calcium, PTH levels and renal function as well as annual bone mineral density scans.

### Parathyroidectomy

The main goals when treating patients with MEN1 associated PHPT are: (i) to obtain normocalcemia, (ii) avoid permanent hypoparathyroidism, and (iii) minimize the number of operations whilst facilitating future surgeries in light of the disease process, which manifests at the germline level causing almost inevitably recurrent hyperfunctioning parathyroid tissue. It is critical to choose the best surgical strategy to achieve optimal results with the aforementioned goals in mind.

Three approaches have been described for parathyroidectomy: (i) less than subtotal parathyroidectomy with removal of <3 glands, (ii) total parathyroidectomy with autotransplantation, and (iii) subtotal parathyroidectomy with removal of 3–3.5 glands. In a less than subtotal parathyroidectomy, <3 parathyroid glands are removed. This has been shown to have higher recurrence rates in shorter time periods compared to the other techniques. In a retrospective review performed at MDACC, Romero et al. studied outcomes in MEN1 pediatric patients undergoing a parathyroidectomy. They found that more than 40% of the patients had recurrent HPT with a median time to recurrence of 5.1 years and this was associated with undergoing a less than subtotal parathyroidectomy. In another retrospective review performed at MDACC it was found that 92% of the patients who underwent a less than subtotal parathyroidectomy had persistent or recurrent hyperparathyroidism at 4 years (17). Additionally, in a retrospective review Nilubol et al. showed persistent hyperparathyroidism of 69 and 20% in patients who underwent a less than subtotal parathyroidectomy of 1–2 and 2.5–3 glands, respectively, compared to 6% in patients who underwent removal of at least 3.5 parathyroids (18). In a retrospective review of total parathyroidectomies with autotransplantation, Tonelli et al. found a recurrence rate of 10% followed with a mean time of 7 years (19).

Hypoparathyroidism is a known risk and possible complication of bilateral neck exploration and subtotal parathyroidectomy. In Nilubol et al.'s review, 10% of the patients who had two or more parathyroids removed had persistent hypoparathyroidism, which was treated with cryopreserved parathyroid autotransplantation in 6% of these patients. This has led the authors to advocate for cryopreservation of parathyroid tissue in these patients (18). In the MDACC review by Lambert et al., none of the patients who underwent a less than subtotal parathyroidectomy had hypoparathyroidism after 10.5 months of follow-up and 7% of the patients who underwent a subtotal parathyroidectomy had hypoparathyroidism after a median follow-up of 3 years (range 10 months to 15 years) (17). Of all three techniques, total parathyroidectomies have the highest risks of permanent hypocalcemia (2, 8, 17) as shown by Lambert et al. (17) and Tonelli et al. (19) where 25% of those cases had permanent hypoparathyroidism. This demonstrates that despite total parathyroidectomy with autotransplantation presenting with the lowest rate of persistent or recurrent hyperparathyroidism in MEN1 patients as indicated previously, it has the highest rate of hypoparathyroidism.

In light of the relative advantages of the subtotal parathyroidectomy procedure, various institutions (8, 17, 20, 21) and experts (2, 4, 5, 18) have favored the removal of 3.5 glands while performing a parathyroidectomy in patients with MEN1 syndrome.

A cervical thymectomy should be performed with any MEN1-related parathyroidectomy as the thymus may contain ectopic or supranumerary parathyroid tissue and it may help remove tissue that has a potential to develop thymic neuroendocrine malignancy. It is estimated that in average only 30–40% of the thymus is removed in a transcervical thymectomy (TCT) (22, 23), which has led its value to be questioned given that thymic carcinoids have still developed in certain patients after transcervical thymic resection (24, 25). Thymic carcinoid in MEN1 patients is estimated to have a prevalence between 2 and 3% (26, 27), and appears to have a male predominance (21). Additionally, the incidence of ectopic or supranumerary thymic parathyroids in these patients is estimated to be 53% (with <4 glands identified during surgery) and 6% (with at least 4 glands identified during surgery) according to Powell et al. (23) This has led Powell et al. to conclude the benefit of TCT to be significant only when <4 parathyroid glands have been identified during surgery. However, at MDACC, we believe there is a dual benefit to performing prophylactic TCT in these patients by removing any ectopic or supranumerary parathyroid glands present in the thymus and decreasing the risk of thymic carcinoid. In a retrospective review performed at MDACC it was found that MEN1 patients who were diagnosed with thymic carcinoid, had been diagnosed with PHPT in average 1 year prior to second diagnosis and none of the patients who underwent a parathyroidectomy had a prophylactic TCT to their knowledge (28). Additionally in a series of 97 MEN1 patients, a Dutch group found no incidence of thymic carcinoid in their patients who underwent a prophylactic TCT during parathyroidectomy with an average follow-up of 8 years (range 0–40 years) (29).

#### Subtotal parathyroidectomy (our selected approach)

The most important decision making with regards to patient management takes place in clinic. Accurate diagnosis of MEN1 syndrome in the preoperative setting is crucial as it will allow appropriate counseling and dictate the correct procedure. This requires confirmative genetic testing or clinical documentation meeting the definition of MEN1 as described previously. Failure to recognize the presence of MEN1 preoperatively is associated with higher rates of persistent PHPT (10, 12).

For our preoperative imaging, we only obtain an ultrasound of the neck. This allows for evaluation of the thyroid gland to rule out any concomitant pathology that may warrant intervention. We do not advocate for additional imaging. Localization studies, such as functional nuclear medicine assessment are not warranted since all four parathyroid glands will be explored during the operation (5). In their review, Nilubol et al. found that only 7% of ectopic parathyroids were identified by preoperative imaging in their patients while the median number of parathyroids identified by preoperative imaging in their patients was only one gland. The most cost-effective study was ultrasound as it identified 3 of the 4 ectopic parathyroids (7%) identified preoperatively. Additionally in 5% of their patients no hyperfunctioning parathyroid was identified by preoperative imaging. The authors recognize the use of preoperative ultrasound as it can help identify the majority of extrathymic ectopic parathyroids and is more economic. However, they do not advocate the routine use of preoperative imaging in MEN1 patients undergoing bilateral neck exploration and subtotal parathyroidectomy as it will potentially alter the surgical approach in only 7% of the patients (30). Imaging will not add great value (defined as outcome/cost) to our patient management and we agree with the authors, as the cost is not justified for preoperative workup in these patients.

At MDACC, we perform subtotal parathyroidectomy in patients with MEN1 syndrome by removal of 3.5 glands or more, but <4 with the goal of leaving a remnant 1.5–2 times the size of a normal gland or the equivalent: 40–60 mg (a normal gland measures ~30 mg). We also perform bilateral cervical thymectomy. The aggressiveness of this latter aspect of the operation is performed with judgment to ensure that the blood supply to the remnant, if in the inferior position, is not compromised.

##### Description of procedure

*Preoperative planning:* If this is the patient's first neck surgery, we do not use the nerve monitor as it has not been shown to reduce nerve injury (31). The only instance where we use the nerve monitor is in the reoperative cervical procedure where extensive scarring is anticipated or when one of the recurrent laryngeal nerves has been damaged previously. Our goal of nerve monitoring is to ensure the nerve is intact at the conclusion of the case. This allows us to be prepared for cautious scrutiny of the airway at extubation and in the immediate post-operative period. We do not use the nerve monitor to decrease our risk of injury. Even in reoperative necks, the use of intraoperative nerve monitoring has not shown to reduce nerve injury (32). Additionally we do not use intraoperative PTH (IOPTH) in these patients as it will not give us additional information in our management but does considerably increase operative time and cost. In our practice, IOPTH suggests when to stop operating because adequate removal of all hyperfunctioning tissue has occurred and further inspection of the other glands is not necessary, such as in minimally invasive parathyroidectomy for single gland disease. This is not the case in our planned bilateral cervical explorations for known four gland disease as it will not guide the extent of the parathyroidectomy (18). In other institutions, IOPTH is part of their practice even in planned bilateral neck explorations as it may indicate successful or failed removal of all hyperfunctioning tissue (33) (nonetheless there can also exist a false positive decrease in IOPTH despite the presence of other hyperfunctioning tissue). However, as shown by Nilubol et al., having a cutoff for IOPTH, even if high (≥75%) does not exclude persistent PHPT or hypoparahyroidism (34). In our practice we believe that performing IOPTH will not add anything to our surgical planning: we are already planning on finding the four glands, but it will however increase our costs and operative time as mentioned previously, therefore limiting its value.

*Procedure:* In the operating room, the patient is placed in a semi-Fowler position with physiologic hyperextension of the neck.

We proceed to mark the skin for our incision and draw a line two fingerbreadths above the sternum and clavicles in the midline.

**Figure 1:**
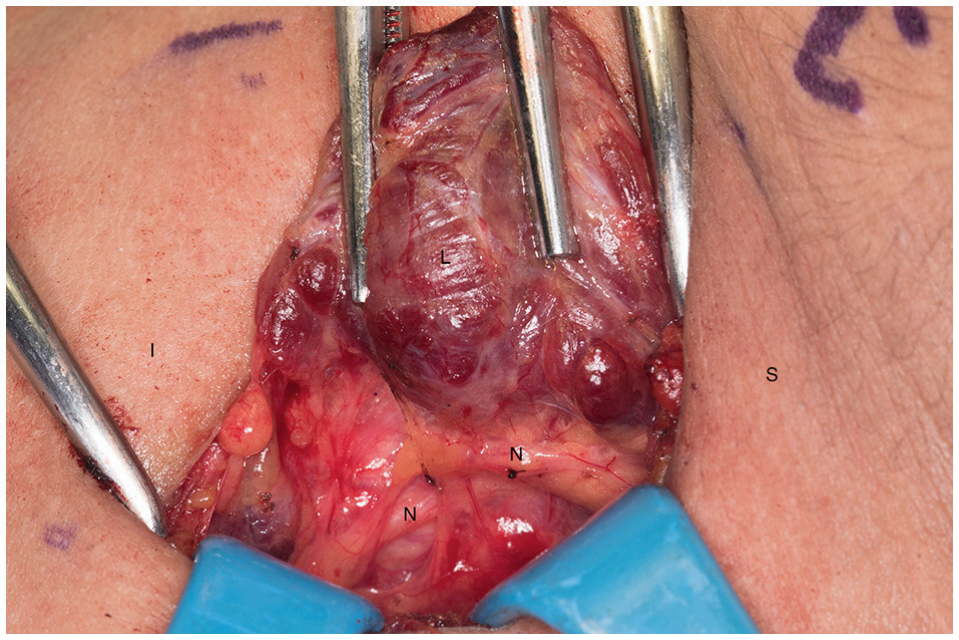
Left recurrent laryngeal nerve and left parathyroids are exposed by performing the “up and over” motion. S, superior; I, inferior; L, left thyroid lobe; N, recurrent laryngeal nerve.

**Figure 2:**
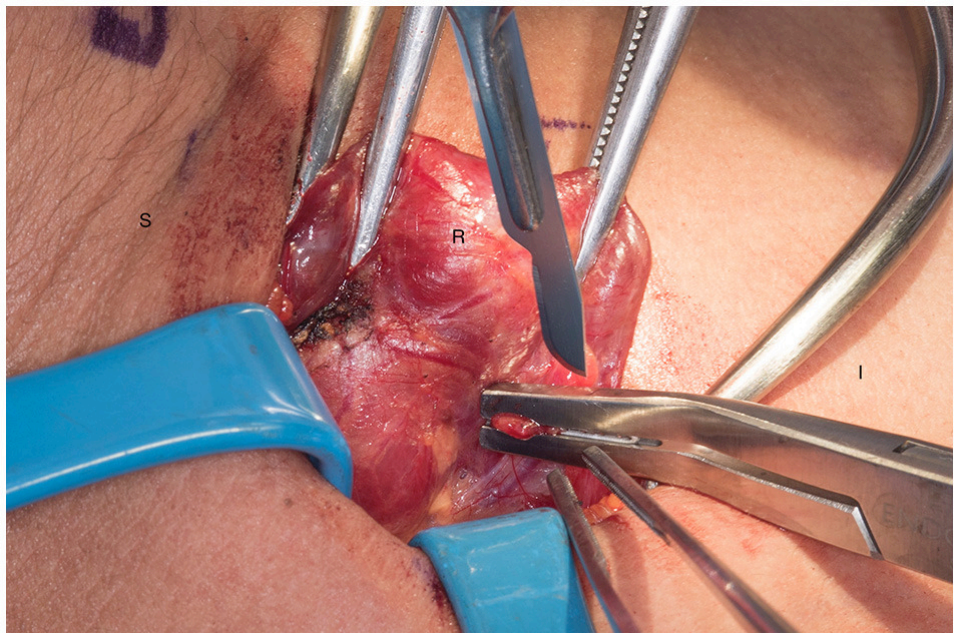
Transection of the remnant with a hemoclip and sharp blade (preferable #10). S, superior, I, inferior, R, right thyroid lobe.

**Figure 3:**
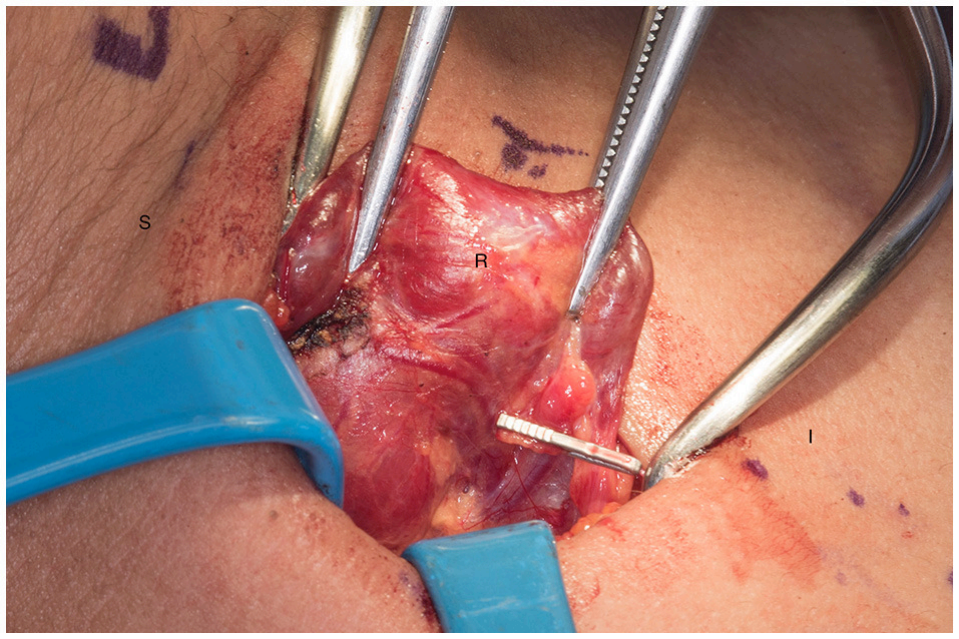
Presence of hemoclip on remnant of the right inferior parathyroid gland. S, superior; I, inferior; R, right thyroid lobe.

**Figure 4:**
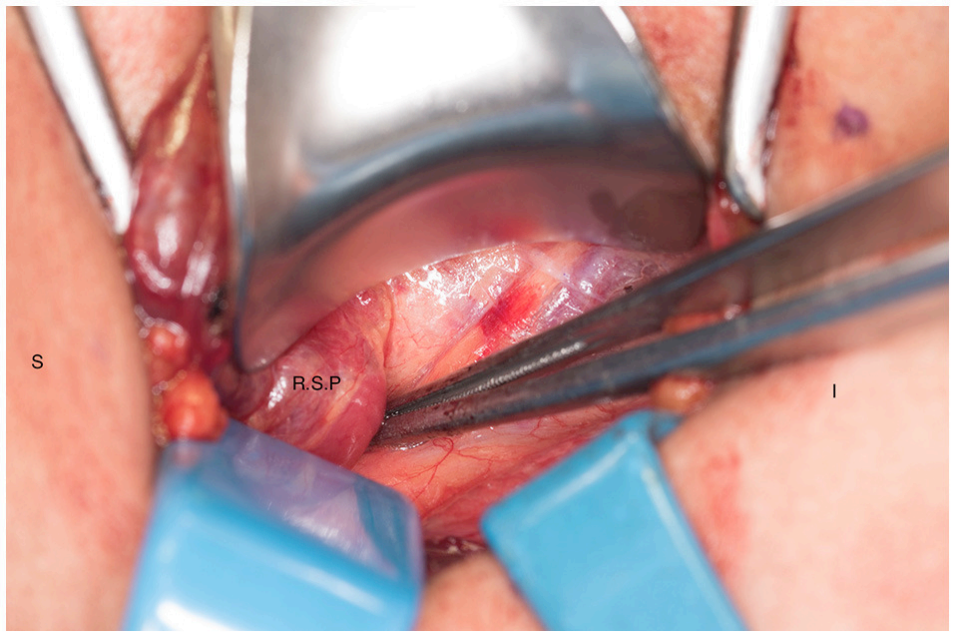
Right superior parathyroid gland is isolated. S, superior; I, inferior; R.S.P, right superior parathyroid.

**Figure 5:**
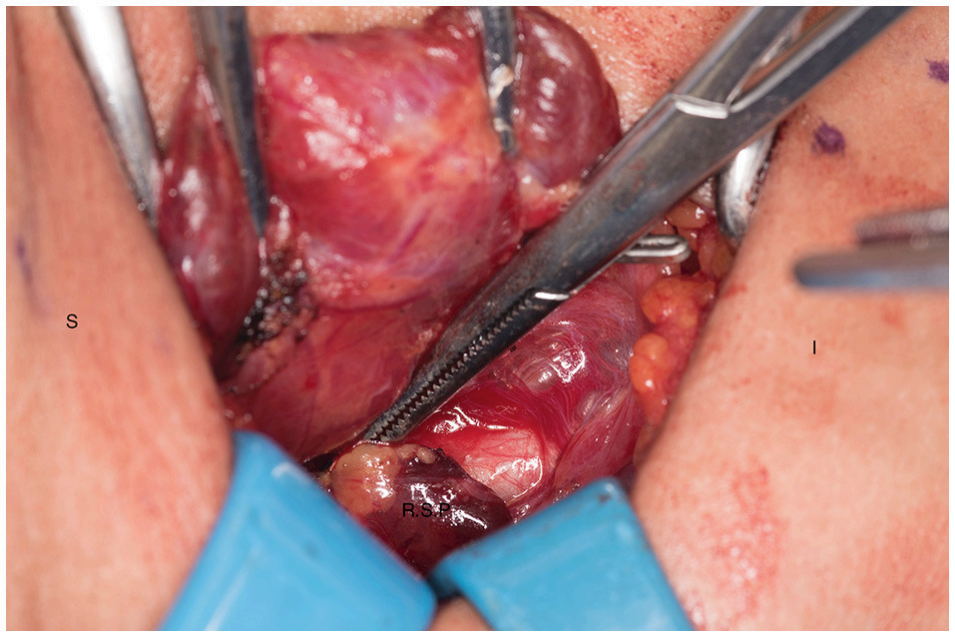
Ischemic right superior parathyroid gland after dissection and being transected. S, superior; I, inferior; R.S.P, right superior parathyroid.

**Figure 6:**
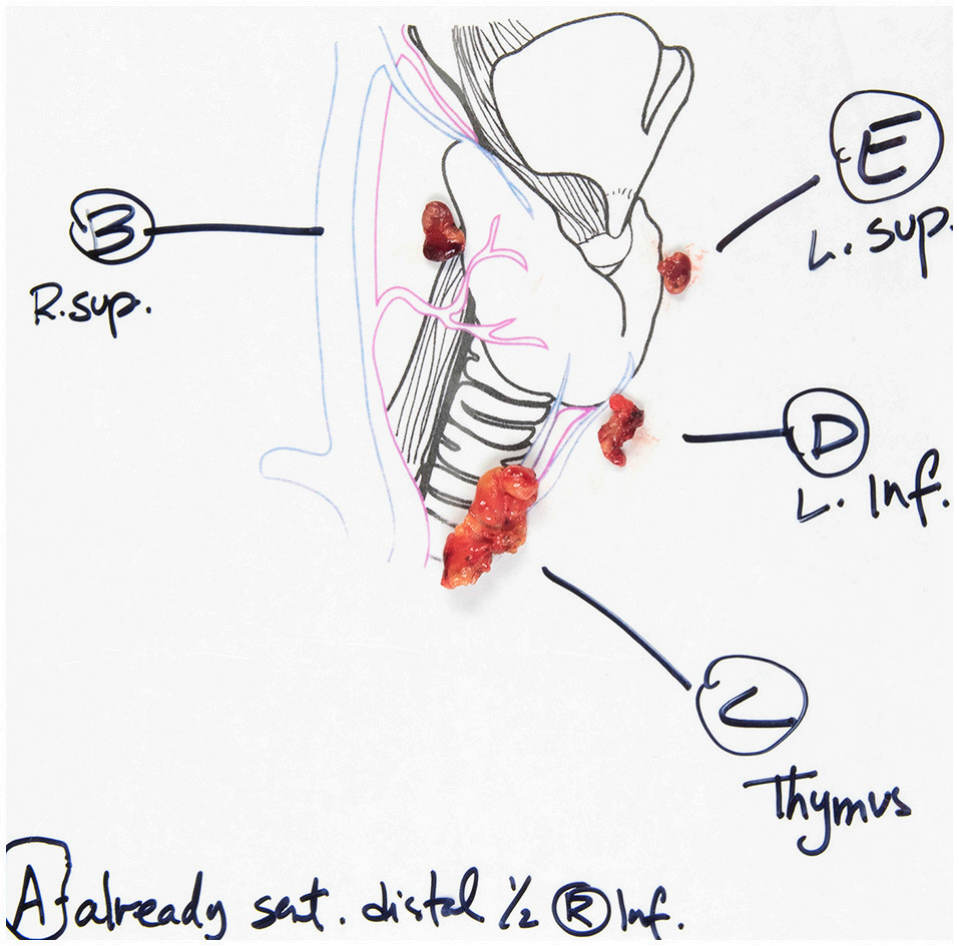
Laminated illustration with our specimens laid in their anatomical position.

We systematically start our procedure on the right side, unless there are unusual circumstances. This ensures consistency with our OR team for labeling of specimens to avoid any confusion. The skin, subcutaneous tissue and platysma are cauterized and divided. The right-sided strap muscles are then identified and retracted medially while the sternocleidomastoid muscle is retracted laterally. The muscles are retracted off of the anterior surface of the thyroid parenchyma. We place two Kochers on the right thyroid lobe, from superior pole and from the inferior pole and retract the gland medially toward the ceiling and away from the operating surgeon in an up and over motion (Figure 1). This will expose the recurrent laryngeal nerve and both parathyroid glands. Once these structures have been identified, we dissect both superior and inferior parathyroid glands with the aid of the bipolar device. During this initial dissection we are extremely cautious to only dissect medial to the glands as not to disrupt the blood supply from the pedicle to ensure chosen remnant viability. We start our resection with crafting of our chosen remnant. Whenever possible we favor the right inferior parathyroid for our remnant as anatomically inferior glands are easiest to re-access in the future if necessary. In our decision making process, we also use size, degree of nodularity, consideration of which gland is the most normal appearing, location of the remnant to the recurrent laryngeal nerve and the positioning relative to the superior aspect of the thymus. If indeed the inferior parathyroid gland meets these qualifications, we place a large Hemoclip across the distal of the right inferior gland and while holding the clip in place, we sharply resect the distal end of the parathyroid with a #10 blade knife. The Hemoclip is left in place to facilitate future identification if necessary (Figures 2, 3). The resected distal specimen is sent to pathology for frozen section review and confirmation that this is indeed hypercellular parathyroid tissue. We then direct our attention to the superior gland and remove it by dividing the pedicle with the bipolar device (Figures 4, 5). All specimens are placed on laminated illustrations of the right and left neck where we lay our specimens to document their positioning. This is photographed for the medical record (Figure 6). By performing our partial resection early on during the procedure we will be able to reassess our remnant's viability after completing bilateral neck explorations, giving it time to manifest ischemic changes if the blood supply was compromised.

**Figure 7:**
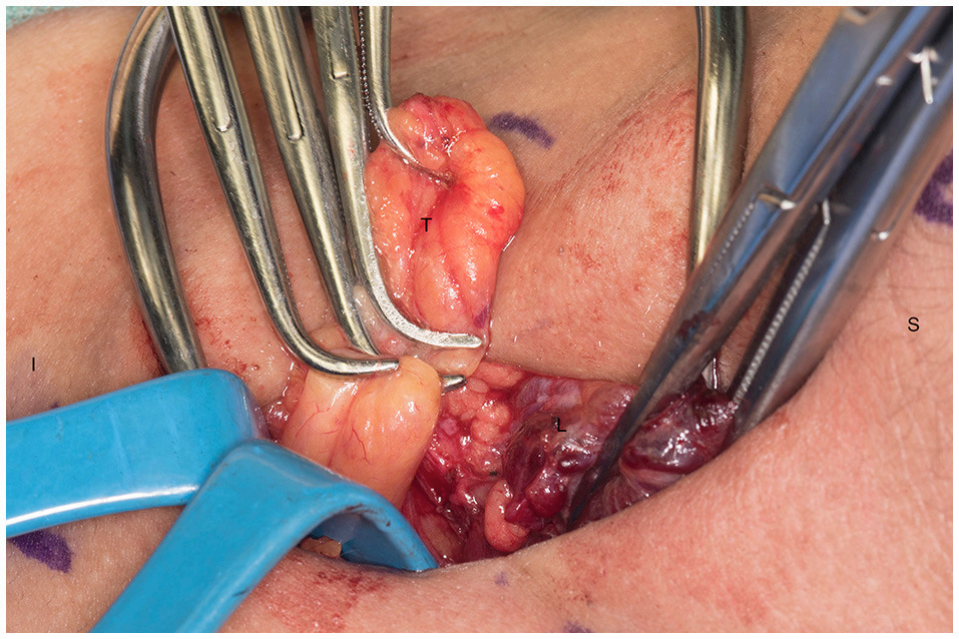
Left cervical thymectomy. S, superior; I, inferior; T, thymus; L, left thyroid lobe.

**Figure 8:**
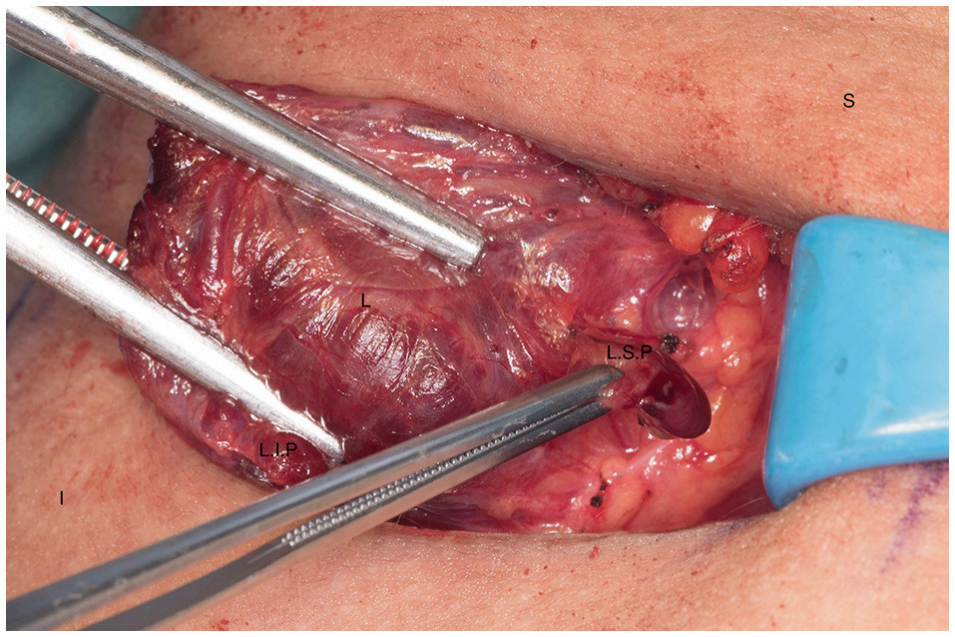
Dissection of the left superior parathyroid gland. S, superior; I, inferior; L.S.P, left superior parathyroid; L.I.P, left inferior parathyroid.

We then proceed toward the left side of the neck and repeat the above dissection and proceed with resection of the thymus first. We retract it meticulously with sequential right angled retractors delivering the tissue from the mediastinum into the neck (Figure 7). We then proceed with grasping the inferior-most aspect of thymic parenchyma that is safely delivered and transect it with Harmonic or electrocautery. This is critical to avoid bleeding. We then complete a symmetric procedure on the right cervical bed if the two aspects of thymus are anatomically separated. In certain instances the positioning of the right inferior parathyroid may put it at risk for devascularization during the thymectomy and we may choose to forego this portion of the procedure. Upon resection of the thymus we inspect the tissue in an *ex-vivo* fashion to confirm the absence of an inferior or supernumerary gland. Prior to resecting the left sided parathyroid glands we return to our right neck to evaluate our remnant and confirm its perfusion and viability. We also confirm hypercellular parathyroid tissue by the pathology frozen section analysis. Having this in process while we dissect the contralateral glands avoids inefficiencies. Once we have confirmed that the parathyroid remnant is viable (at least 15–20 min of operating time has occurred since crafting the residual tissue) we proceed with resection of our left sided parathyroid glands with the bipolar device (Figure 8). If our remnant is not well-perfused or we are concerned of its viability, we proceed with crafting a new remnant from one of the left sided glands. All removed glands are sent for pathologic review. The neck incision is then closed in the usual fashion.

One of the challenges can be identification of the parathyroid glands, particularly if they are pathologic and have displaced themselves due to their weight. We use a systematic approach following embryological principles to identify the parathyroids which we will discuss.

##### Identification of the parathyroid glands

We have previously described an alphabetical nomenclature system to better communicate and standardize exploration of parathyroid glands. Superior glands will be type A, B, or C and inferior glands will be type E or F. A type D gland can be either and a type G gland is intrathyroidal.

Superior parathyroid glands are lateral to the recurrent laryngeal nerve. We look for them on the posterior thyroid capsule (Type A), in the tracheoesophageal groove behind the thyroid lobe (Type B) and inferiorly toward the clavicle (Type C). We also inspect along the course of the recurrent laryngeal nerve (Type D) (many glands are within 1 cm of the RLN). Embryologically inferior glands reside near the external edge of the inferior thyroid pole (Type E) or have fallen inferiorly into the thyrothymic ligament or the thymus (Type F). The preoperative ultrasound examination may support or refute the possibility of an intrathyroidal gland (Type G) which is a very rare instance (35).

By following this systematic approach, all eutopic parathyroid glands should be accounted for.

## Post-operative management and follow-up

Two hours after the subtotal parathyroidectomy in these patients, we draw a calcium level and an intact PTH. We repeat these values around 5 p.m. of the day of surgery and at 5 a.m. on post-operative day 1. PTH has a 2–4 min half-life, which is why we order blood work twice on post-operative day 0, to monitor the PTH trend and observe if it reacts to a decrease in serum calcium levels. If our patients present with signs of hypocalcemia, such as perioral numbness and finger paresthesias we provide them with calcium carbonate tablets which usually relieves their symptoms. If the patient's PTH does not trend upwards and keeps decreasing on our laboratory values, or our calcium is significantly low post-operatively we will consider placing the patient on supplemental calcium until their post-operative visit and reassess. We do not systematically place our patients on calcium supplement as we want to stimulate the remnant in producing PTH and giving the patient oral calcium could inhibit this stimulation. Patients may have transient hypoparathyroidism but it is not defined as permanent until 6 months post-operatively with persistent low PTH levels. We revisit with our patients 2 weeks after surgery. We see them again 6 months after surgery to monitor their PTH and calcium.

Following parathyroidectomy, we perform annual surveillance with serum measurements of intact PTH and calcium levels. One year after surgery and then every 2 years, we order a bone mineral density scan focusing on the distal forearm, lumbar spine and femoral neck. Due to its lower bone turnover, the distal radius is the earliest and most affected bone in sporadic PHPT (36) as well as in the MEN1 subset (15). Therefore, post-operatively, the bone mineral density of the lumbar spine and femoral neck are the most reliable to evaluate bone health as the distal radius may take longer (18 months) to recover and does not always recover completely (36, 37).

## Recurrence

Recurrence is defined as recurrent hypercalcemia with inappropriately normal or elevated serum intact PTH 6 months after surgery, when normocalcemia and normal intact PTH levels had been achieved post-operatively. If this happens in the first 6 months after surgery it is considered persistent PHPT and not recurrent (11). It is estimated that 40–60% of MEN1 patients with PHPT will recur 10–12 years after their initial parathyroidectomy (2) with nearly half of the patients recurring by 15 years (20) compared to about only 6% in patients with single parathyroid gland disease after 10 years (38). When we perform the initial parathyroidectomy in these patients, we know they will require a re-operation in most cases, particularly if they are young (third to fourth decade or younger) (10). Therefore, it is inappropriate to consider these patients cured after their initial procedure.

We follow-up these patients on an annual basis, and the diagnosis of recurrence is done biochemically. We see a progressive increase in their calcium levels with concomitant inappropriate intact PTH levels. Our decision to re-operate in these patients is more conservative. The risk of complications is higher in re-operative cervical exploration (12–33% compared to 2% in non-operated neck (19, 39) and the decision to re-operate and which procedure to perform is individualized for each patient.

The decision to proceed or not with reoperative cervical intervention in our patients with recurrent hypercalcemia is multifactorial. First we confirm the diagnosis biochemically. We then perform non-invasive imaging studies, such as an ultrasound of the neck and a Tc99m sestamibi-SPECT (Mibi/SPECT) in order to localize an additional parathyroid gland that could have been missed during the first surgery or localize the remnant, particularly if the first procedure was performed at an outside hospital. It is important to obtain prior pathology records and operative reports. All patients with prior cervical operation require an evaluation of their vocal cord function preoperatively and this will also influence our decision to proceed with intervention or not. We re-operate on patients who have symptoms of PHPT or significant hypercalcemia (above 11 mg/dL). If our patients have significant comorbidities increasing their American Society of Anesthesiologists Score, and have increased risk of morbidity, medical therapy, such as calcimimetics is an alternative to a second cervical operation (4, 40).

In re-operative cervical exploration we use the nerve monitor, as we expect extensive scarring and particularly if there is a known unilateral cord paralysis. This allows us to ensure the nerve is intact at the end of the case and if it appears to have been damaged, we cautiously monitor the airway during extubation and in the immediate post-operative period. Additionally we use IOPTH as this informs us of when to stop our cervical exploration. This practice correlates with Keutgen et al.'s recommendation when they reviewed their data in MEN1 patients with recurrent or persistent HPT after a bilateral neck exploration (10).

Patient positioning is as described in the earlier portion of this manuscript. If our localization studies did not suggest the presence of an additional gland apart from the remnant, we perform a focused parathyroidectomy, such as a minimally invasive approach if deemed safe or a unilateral neck exploration to localize the remnant. Our incision is performed as described above and our baseline IOPTH is drawn at the time of incision. Assuming our remnant is on the right side as we tend to leave it and following our nomenclature mentioned previously, we direct our attention to the right neck. Due to the scarring we favor a lateral approach and separate the strap muscles off of the thyroid lobe and retract them laterally. The thyroid lobe is retracted medially as described previously. Once the remnant and the recurrent laryngeal nerve have been localized and dissected using the bipolar device, we perform a debulking of the remnant with our hemoclip technique, leaving here again, a remnant 1.5–2 times the size of a normal gland. We send the transected tissue to pathology for frozen confirmation and draw an IOPTH after 5 and 10 min. We expect a 50% decrease in our IOPTH compared to baseline. If this drop in IOPTH is not obtained we may decide to resect additional tissue from our remnant or perform an autotransplantation into the brachioradialis muscle. If our imaging suggested the presence of an additional parathyroid gland we start our procedure targeting that specific gland and then direct our attention to the remnant if our IOPTH is persistently elevated.

If we encounter significant scarring and a difficult dissection, we may decide to complete a total parathyroidectomy with autotransplantation of the remnant into the nondominant brachioradialis muscle. As discussed, patients with MEN1 syndrome are at a high risk of lifetime recurrence of PHPT and we know they may require additional debulking of the remnant. If we estimate that a future cervical repeat re-exploration would have unacceptable high morbidity risks, we favor an autotransplantation.

## Conclusion

PHPT in MEN1 patients is a difficult disease that requires strategic planning with regards to management. Thorough knowledge of therapeutic options and understanding of the long-term outcomes is critical to the optimal care of these patients. It is a constant balance between the benefits and the risks we subject our patients to, knowing that the mutation in the *MEN1* gene predisposes the remaining parathyroid tissue to become hypercellular. With all the disease factors taken into consideration we perform subtotal parathyroidectomy in MEN1 patients with PHPT, knowing that they will present with recurrent PHPT within 15 years of their first operation. The lifelong consequences of making a patient aparathyroid are devastating and we prefer to reoperate on a patient in 15 years rather than increasing their risk of hypoparathyroidism by removing all four glands at the first operation through a total parathyroidectomy with autotransplantation. Hopefully by doing so, we allow our patients to be euparathyroid for an additional 15 years of their life, if not more.

## Author contributions

PN wrote the article, did the research, discussed the topic with NP and reviewed every draft and made changes according to the reviewers' comments for each draft. EA reviewed and corrected the first and second draft of the article. JZ reviewed and corrected the first and second draft of the article. NP coordinated the writing of the article, reviewed every draft, gave input on article content, and what needed to be included.

### Conflict of interest statement

The authors declare that the research was conducted in the absence of any commercial or financial relationships that could be construed as a potential conflict of interest.

## References

[1] Perrier ND. From initial description by wermer to present-day MEN1: what have we learned? World J Surg. (2018)42:1031–5. 10.1007/s00268-017-4435-3 29383428

[2] Thakker RV Newey PJ Walls GV Bilezikian J Dralle H Ebeling PR. Clinical practice guidelines for multiple endocrine neoplasia type 1 (MEN1). J Clin Endocrinol Metab. (2012)97:2990–3011. 10.1210/jc.2012-1230 22723327

[3] Marini F Falchetti A Del Monte F Carbonell Sala S Gozzini A Luzi E. Multiple endocrine neoplasia type 1. Orphanet J Rare Dis. (2006) 1:38. 10.1186/1750-1172-1-38 17014705 PMC1594566

[4] Kraimps JL Beaulieu A Donatini G Familial hyperparathyroidism in multiple endocrine neoplasia syndromes. In: Clark OH Duh Q-Y Kebebew E editors. Textbook of Endocrine Surgery, 3rd ed Philadelphia, PA: The Health Sciences Publisher (2016). p. 831–6.

[5] Brandi ML Gagel RF Angeli A Bilezikian JP Beck-Peccoz P Bordi C. Guidelines for diagnosis and therapy of MEN type 1 and type 2. J Clin Endocrinol Metab. (2001)86:5658–71. 10.1210/jcem.86.12.8070 11739416

[6] Romero Arenas MA Morris LF Rich TA Cote GJ Grubbs EG Waguespack SG Preoperative multiple endocrine neoplasia type 1 diagnosis improves the surgical outcomes of pediatric patients with primary hyperparathyroidism. J Pediatr Surg. (2014)49:546–50. 10.1016/j.jpedsurg.2013.11.059 24726110

[7] Kouvaraki MA Greer M Sharma S Beery D Armand R Lee JE. Indications for operative intervention in patients with asymptomatic primary hyperparathyroidism: practice patterns of endocrine surgery. Surgery (2006)139:527–34. 10.1016/j.surg.2005.09.006 16627063

[8] Schreinemakers JM Pieterman CR Scholten A Vriens MR Valk GD Rinkes IH. The optimal surgical treatment for primary hyperparathyroidism in MEN1 patients: a systematic review. World J Surg. (2011)35:1993–2005. 10.1007/s00268-011-1068-9 21713580

[9] Lee CH Tseng LM Chen JY Hsiao HY Yang AH. Primary hyperparathyroidism in multiple endocrine neoplasia type 1: individualized management with low recurrence rates. Ann Surg Oncol. (2006)13:103–9. 10.1245/ASO.2006.12.009 16378158

[10] Keutgen XM Nilubol N Agarwal S Welch J Cochran C Marx SJ. Reoperative surgery in patients with multiple endocrine neoplasia Type 1 associated primary hyperparathyroidism. Ann Surg Oncol. (2016)23:701–7. 10.1245/s10434-016-5467-x 27464610 PMC6415766

[11] Wilhelm SM Wang TS Ruan DT Lee JA Asa SL Duh QY. The American Association of endocrine surgeons guidelines for definitive management of primary hyperparathyroidism. JAMA Surg. (2016)151:959–68. 10.1001/jamasurg.2016.2310 27532368

[12] Tonelli F Giudici F Cavalli T Brandi ML. Surgical approach in patients with hyperparathyroidism in multiple endocrine neoplasia type 1: total versus partial parathyroidectomy. Clinics (Sao Paulo) (2012) 67(Suppl. 1):155–60. 10.6061/clinics/2012(Sup01)26 22584722 PMC3328832

[13] Burgess JR David R Greenaway TM Parameswaran V Shepherd JJ. Osteoporosis in multiple endocrine neoplasia type 1: severity, clinical significance, relationship to primary hyperparathyroidism, and response to parathyroidectomy. Arch Surg. (1999)134:1119–23. 10522858 10.1001/archsurg.134.10.1119

[14] Silva AM Vodopivec D Christakis I Lyons G Wei Q Waguespack SG. Operative intervention for primary hyperparathyroidism offers greater bone recovery in patients with sporadic disease than in those with multiple endocrine neoplasia type 1-related hyperparathyroidism. Surgery (2017)161:107–115. 10.1016/j.surg.2016.06.065 27842919

[15] Lourenco DM Jr Coutinho FL Toledo RA Gonçalves TD Montenegro FLM ToledoIet SPAal. Biochemical, bone and renal patterns in hyperparathyroidism associated with multiple endocrine neoplasia type 1. Clinics (Sao Paulo) (2012) 67(Suppl. 1):99–108. 10.6061/clinics/2012(Sup01)17 22584713 PMC3329618

[16] Giusti F Tonelli F Brandi ML. Primary hyperparathyroidism in multiple endocrine neoplasia type 1: when to perform surgery? Clinics (Sao Paulo) (2012) 67(Suppl. 1):141–4. 10.6061/clinics/2012(Sup01)23 22584719 PMC3328829

[17] Lambert LA Shapiro SE Lee JE Perrier ND Truong M Wallace MJ. Surgical treatment of hyperparathyroidism in patients with multiple endocrine neoplasia type 1. Arch Surg. (2005)140:374–82. 10.1001/archsurg.140.4.374 15841561

[18] Nilubol N Weinstein LS Simonds WF Jensen RT Marx SJ Kebebew E. Limited parathyroidectomy in multiple endocrine neoplasia type 1-associated primary hyperparathyroidism: a setup for failure. Ann Surg Oncol. (2016)23:416–23. 10.1245/s10434-015-4865-9 26542588

[19] Tonelli F Marcucci T Fratini G Tommasi MS Falchetti A Brandi ML. Is total parathyroidectomy the treatment of choice for hyperparathyroidism in multiple endocrine neoplasia type 1? Ann Surg. (2007)246:1075–82. 10.1097/SLA.0b013e31811f4467 18043113

[20] Arnalsteen LC Alesina PF Quiereux JL Farrel SG Patton FN Carnaille BM. Long-term results of less than total parathyroidectomy for hyperparathyroidism in multiple endocrine neoplasia type 1. Surgery (2002)132:1119–24; discussion 1124–5. 10.1067/msy.2002.128607 12490864

[21] Norton JA Venzon DJ Berna MJ Alexander HR Fraker DL Libutti SK. Prospective study of surgery for primary hyperparathyroidism (HPT) in multiple endocrine neoplasia-type 1 and Zollinger-Ellison syndrome: long-term outcome of a more virulent form of HPT. Ann Surg. (2008)247:501–10. 10.1097/SLA.0b013e31815efda5 18376196 PMC2717476

[22] Montenegro FL Lourenco DM Jr Tavares MR Arap SS Nascimento CP Jr Massoni Neto LM. Total parathyroidectomy in a large cohort of cases with hyperparathyroidism associated with multiple endocrine neoplasia type 1: experience from a single academic center. Clinics (Sao Paulo) (2012) 67(Suppl. 1):131–9. 10.6061/clinics/2012(Sup01)22 22584718 PMC3328834

[23] Powell AC Alexander HR Pingpank JF Steinberg SM Skarulis M Bartlett DL. The utility of routine transcervical thymectomy for multiple endocrine neoplasia 1-related hyperparathyroidism. Surgery (2008)144:878–83; discussion 883–4. 10.1016/j.surg.2008.08.031 19040992 PMC2625284

[24] Burgess JR Giles N Shepherd JJ Malignant thymic carcinoid is not prevented by transcervical thymectomy in multiple endocrine neoplasia type 1. Clin Endocrinol. (2001)55:689–93. 10.1046/j.1365-2265.2001.01348.x 11894982

[25] Gibril F Chen YJ Schrump DS Vortmeyer A Zhuang Z Lubensky IA. Prospective study of thymic carcinoids in patients with multiple endocrine neoplasia type 1. J Clin Endocrinol Metab. (2003)88:1066–81. 10.1210/jc.2002-021314 12629087

[26] Goudet P Murat A Cardot-Bauters C Emy P Baudin E du Boullay Choplin H. Thymic neuroendocrine tumors in multiple endocrine neoplasia type 1: a comparative study on 21 cases among a series of 761 MEN1 from the GTE (Groupe des Tumeurs Endocrines). World J Surg. (2009)33:1197–207. 10.1007/s00268-009-9980-y 19294466

[27] Ferolla P Falchetti A Filosso P Tomassetti P Tamburrano G Avenia N. Thymic neuroendocrine carcinoma (carcinoid) in multiple endocrine neoplasia type 1 syndrome: the Italian series. J Clin Endocrinol Metab. (2005)90:2603–9. 10.1210/jc.2004-1155 15713725

[28] Christakis I Qiu W Silva Figueroa AM Hyde S Cote GJ Busaidy NL. Clinical features, treatments, and outcomes of patients with thymic carcinoids and multiple endocrine neoplasia Type 1 syndrome at MD Anderson cancer center. Horm Cancer (2016)7:279–87. 10.1007/s12672-016-0269-y 27311764 PMC10355947

[29] de Laat JM Pieterman CR van den Broek MF Twisk JW Hermus AR Dekkers OM Natural course and survival of neuroendocrine tumors of thymus and lung in MEN1 patients. J Clin Endocrinol Metab. (2014)99:3325–33. 10.1210/jc.2014-1560 24915123

[30] de Laat JM Pieterman CR van den Broek MF Twisk JW Hermus AR Dekkers OM Preoperative localizing studies for initial parathyroidectomy in MEN1 syndrome: is there any benefit? World J Surg. (2012)36:1368–74. 10.1007/s00268-012-1451-1 22350475

[31] Mourad M Kadakia S Jategaonkar A Gordin E Ducic Y. Intraoperative nerve monitoring during parathyroid surgery: the fort worth experience. Head Neck (2017)39:1662–4. 10.1002/hed.2481 28467621

[32] Yarbrough DE Thompson GB Kasperbauer JL Harper CM Grant CS. Intraoperative electromyographic monitoring of the recurrent laryngeal nerve in reoperative thyroid and parathyroid surgery. Surgery (2004)136:1107–15. 10.1016/j.surg.2004.06.040 15657563

[33] Tonelli F Spini S Tommasi M Gabbrielli G Amorosi A Brocchi A. Intraoperative parathormone measurement in patients with multiple endocrine neoplasia type I syndrome and hyperparathyroidism. World J Surg. (2000)24:556–62; discussion 562–3. 10.1007/s002689910091 10787076

[34] Nilubol N Weisbrod AB Weinstein LS Simonds WF Jensen RT Phan GQ. Utility of intraoperative parathyroid hormone monitoring in patients with multiple endocrine neoplasia type 1-associated primary hyperparathyroidism undergoing initial parathyroidectomy. World J Surg. (2013)37:1966–72. 10.1007/s00268-013-2054-1 23722465 PMC8369518

[35] Perrier ND Edeiken B Nunez R Gayed I Jimenez C Busaidy N. A novel nomenclature to classify parathyroid adenomas. World J Surg. (2009)33:412–6. 10.1007/s00268-008-9894-0 19148701

[36] Marcocci C Cianferotti L Cetani F. Bone disease in primary hyperparathyrodism. Ther Adv Musculoskelet Dis. (2012)4:357–68. 10.1177/1759720X12441869 23024712 PMC3458615

[37] Coutinho FL Lourenco DM Jr Toledo RA Montenegro FLM ToledoIet SPA. Post-surgical follow-up of primary hyperparathyroidism associated with multiple endocrine neoplasia type 1. Clinics (Sao Paulo) (2012) 67(Suppl. 1):169–72. 10.6061/clinics/2012(Sup01)28 22584724 PMC3328812

[38] Schneider DF Mazeh H Chen H Sippel RS. Predictors of recurrence in primary hyperparathyroidism: an analysis of 1386 cases. Ann Surg. (2014)259:563–8. 10.1097/SLA.0000000000000207 24263316 PMC4250051

[39] Karakas E Müller HH Schlosshauer T Rothmund M Bartsch DK. Reoperations for primary hyperparathyroidism–improvement of outcome over two decades. Langenbecks Arch Surg. (2013)398:99–106. 10.1007/s00423-012-1004-y 23001050

[40] Giusti F Cianferotti L Gronchi G Cioppi F Masi L Faggiano A. Cinacalcet therapy in patients affected by primary hyperparathyroidism associated to Multiple Endocrine Neoplasia Syndrome type 1 (MEN1). Endocrine (2016)52:495–506. 10.1007/s12020-015-0696-5 26224587

